# Histopathological Spectrum of a Calcifying Odontogenic Cyst With Uncommon Findings

**DOI:** 10.1155/crid/3651570

**Published:** 2026-01-21

**Authors:** Saraswoti Khadka, Toniya Raut, Neetu Jain, Shashi Keshwar, Ashish Shrestha, Mehul Rajesh Jaisani, Khushbu Adhikari

**Affiliations:** ^1^ Department of Oral Pathology, College of Dental Surgery, B.P. Koirala Institute of Health Sciences, Dharan, Nepal, bpkihs.edu; ^2^ Department of Oral and Maxillofacial Surgery, College of Dental Surgery, B.P. Koirala Institute of Health Sciences, Dharan, Nepal, bpkihs.edu; ^3^ Department of Periodontology and Oral Implantology, College of Dental Surgery, B.P. Koirala Institute of Health Sciences, Dharan, Nepal, bpkihs.edu

**Keywords:** calcifying odontogenic cyst, dentinoid material, ghost cells, odontogenic cyst

## Abstract

Calcifying odontogenic cyst (COC) is an uncommon lesion accounting for less than 1% of all odontogenic cysts. This pathology commonly affects the anterior jaw and is prevalent in the second to third decades of life, with no sex predilection. The lesion presents with a variable clinical and radiographic presentation, which can simulate other common jaw pathologies. Therefore, histopathological confirmation stands to be diagnostic in COC. The fifth edition classification for jaw tumors of the head and neck by the World Health Organization has summarized the essential diagnostic criteria of COC as a jaw cyst occurring in the second to third decades of life with a strong predilection for the anterior mandible and histopathologically characterized by cystic architecture with numerous ghost cells. Here, we present three histopathologically confirmed cases of COC of the mandible, clinically suspected for ameloblastoma, lateral periodontal cyst, and radicular cyst, with emphasis on the characteristics, along with the possible diversity that the lesion can show in its histopathological sections.

## 1. Introduction

Over the years, various terminologies have been used to describe calcifying odontogenic cysts (COCs). The cyst was first described and named by Robert J. Gorlin in 1962 [1], after which it was synonymously known as the Gorlin cyst [2]. The first (1971) and second (1992) editions of the World Health Organization (WHO) classification for head and neck tumors classified this lesion as a cystic lesion without adequate clarification on its neoplastic potential. In the third (2005) edition, the lesion was reclassified as a benign cystic neoplasm with a change in the nomenclature to “calcifying cystic odontogenic tumor.” Until the fourth (2017) edition of the WHO classification, COC faced a dispute on its identity as a cystic or a neoplastic growth. However, the fourth edition ended the controversies and clearly defined COC as a unicystic lesion lined by ameloblastomatous epithelium containing focal accumulation of ghost cells. It was further explained that the ghost cell and ameloblastomatous proliferation are seen in the luminal aspect but are absent or minimal in the mural aspect [2, 3]. The latest fifth (2022) edition forwarded essential features of COC as cystic architecture with the presence of ghost cells. The definition was reformed as a developmental odontogenic cyst characterized histologically by ghost cells, which often calcify, while the phrase “ameloblastomatous epithelium lining” was removed and presented as a desirable feature, not an essential [4, 5]. Here, we report the three cases with uncommon age of presentation, that is, in the fourth, fifth, and seventh decades of life. In contrast, only one case met the histopathological both essential and desired criteria as defined in the fifth edition. All three presented cases exhibited extensive deposition of dentinoid material and ghost cells in the cystic lumen. The cases showed uncommon histopathological diversity, including multinucleated giant cells, melanin pigments within epithelium and ghost cells, and cholesterol cleft.

## 2. Case Presentations (Table 1)

**Table 1:** Clinical findings of reported cases.

**SN**	**Age/gender**	**Duration**	**Site**	**Size (cm** ^**2**^ **)**	**Chief complaint**	**Radiological finding**	**Clinical diagnosis**	**Treatment**	**Follow-up (F/U)**
1	76 Y/F	2.5 years	Mandible (34–44)	5 × 4	Swelling	Unilocular radiolucency	Ameloblastoma	Incisional biopsy	Loss follow‐up
2	47 Y/F	1 year	Mandible (33–34)	1.5 × 1	Swelling	Unilocular radiolucency	Lateral periodontal cyst	Enucleation	Regular F/U (6M‐1 Y)
3	55 Y/F	3 months	Mandible (31–33)	2.5 × 2	Swelling	Unilocular radiolucency	Radicular cyst	Enucleation	Regular F/U (6M‐1 Y)

## 3. Histopathological Presentation

### 3.1. Case 1

On macroscopy, the specimen size was 16 × 14 × 10 mm^3^ and sectioned into four bits.

**Figure 1:**
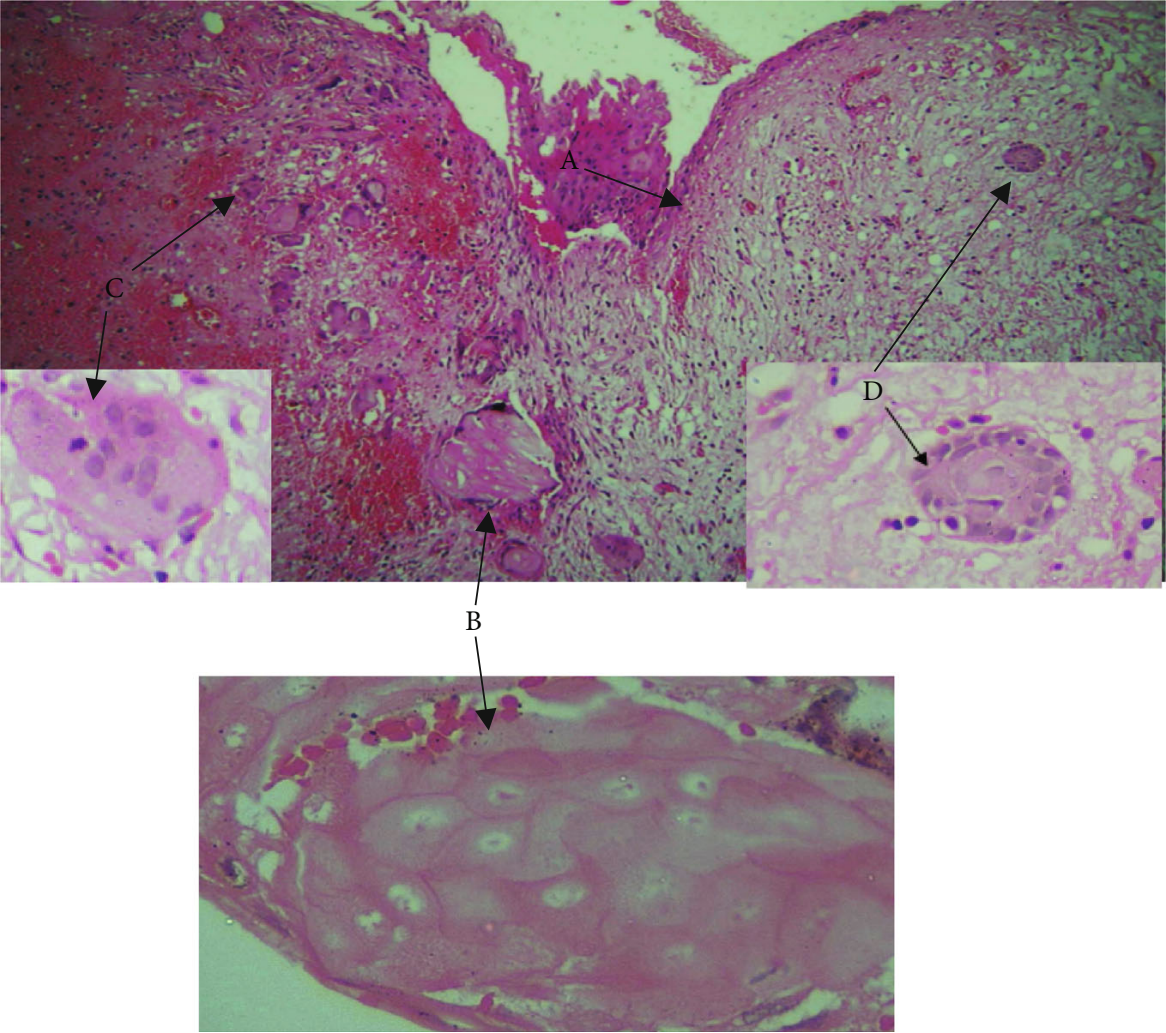
Histopathology of Case 1. Arrows showing (A) cystic lining (10× H&E), (B) ghost cells (10× H&E, 40× H&E), (C) multinucleated giant cell (10×, inset 40× H&E), and (D) odontogenic cell rest (10×, inset 40× H&E).

**Figure 2:**
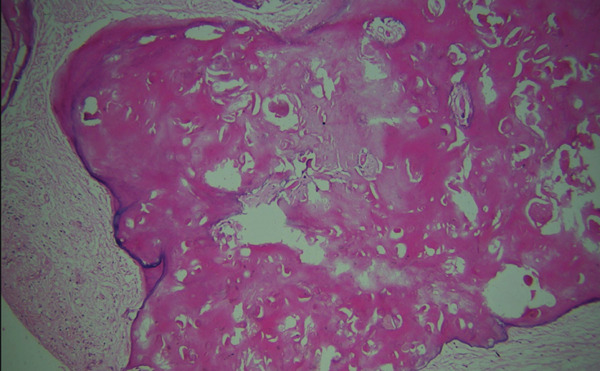
Histopathology of Case 1 showing extensive dentinoid within the cystic lumen (10×, H&E).

On microscopy, the hematoxylin and eosin (H&E)–stained section showed a cystic lesion with a fibrous capsule and a focal area of lining of odontogenic nonkeratinized epithelium (Figure 1A). Underlying connective tissue was fibrocellular, showing sheets of ghost cells with few strands and islands of epithelium (Figure 1B). Numerous giant cells (Figure 1C) were seen adjacent to ghost cells and odontogenic cells (Figure 1D). An extensive mass of eosinophilic dentin‐like structure was also evident within the lumen (Figure 2).

### 3.2. Case 2

Macroscopically, the specimen size was 15 × 12 × 5 mm^3^ on maximum dimension and sectioned into two bits.

**Figure 3:**
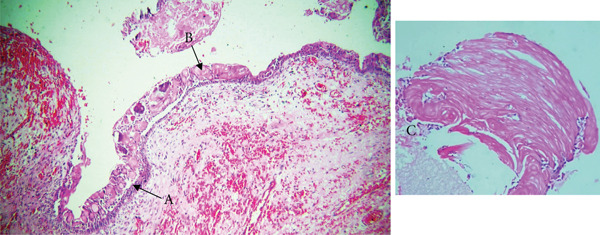
Histological section of Case 2. Arrows showing (A) cystic cavity lined by ameloblast‐like cells (10× H&E), (B) ghost cells (10× H&E), and (C) ghost cells in sheets characterized by eosinophilic cytoplasm and absence of nuclei (40× H&E).

**Figure 4:**
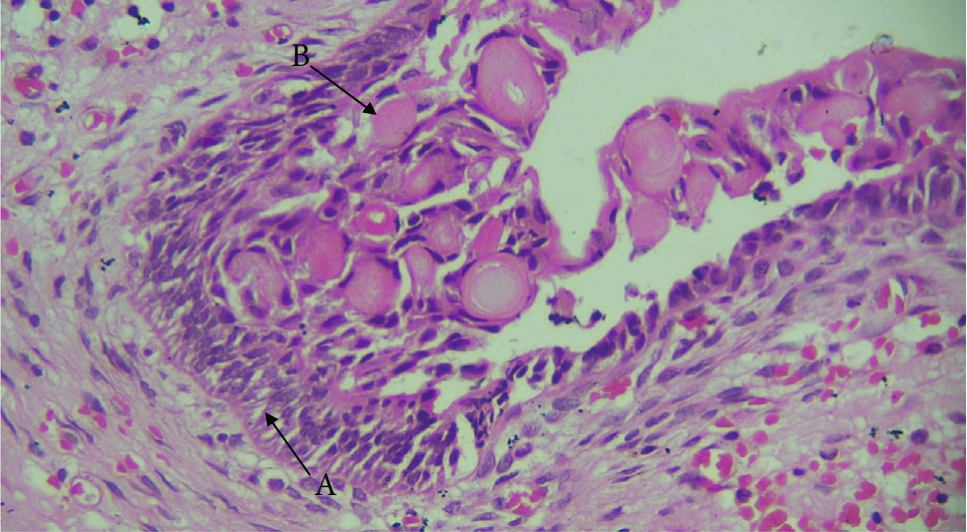
Histological section of Case 2. Arrows showing (A) cystic cavity lined by ameloblast‐like cells (40× H&E) and (B) ghost cells characterized by eosinophilic cytoplasm and absence of nuclei (40× H&E).

**Figure 5:**
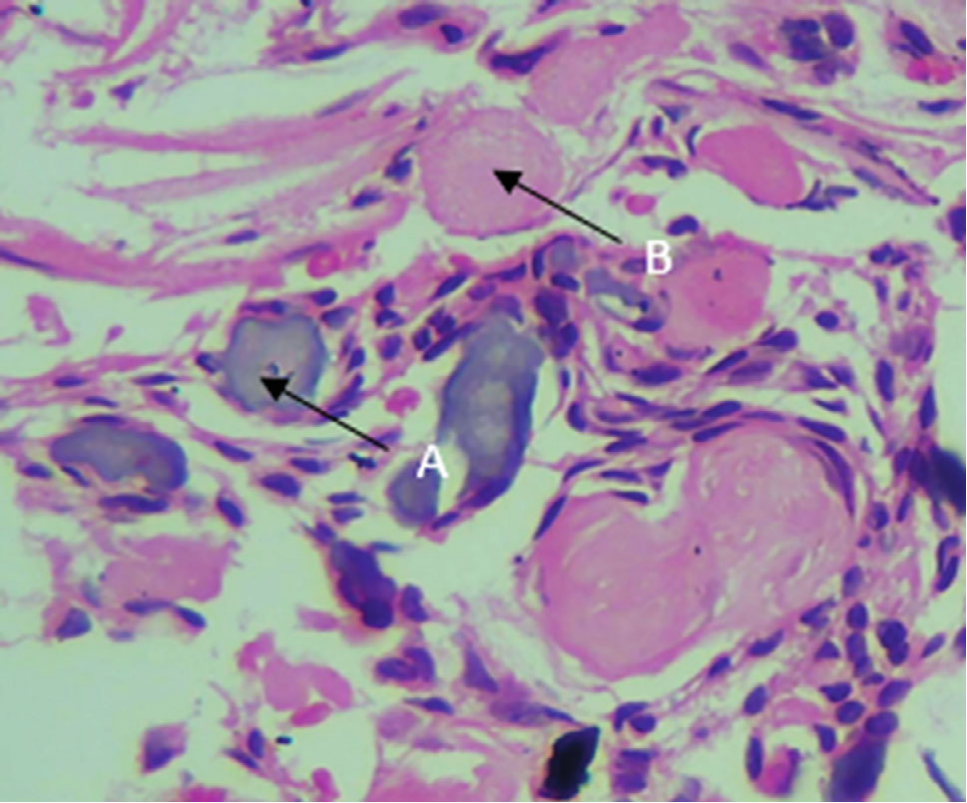
Histological section of Case 2. Arrows showing (A) basophilic calcification (40× H&E) and (B) ghost cells (40× H&E).

**Figure 6:**
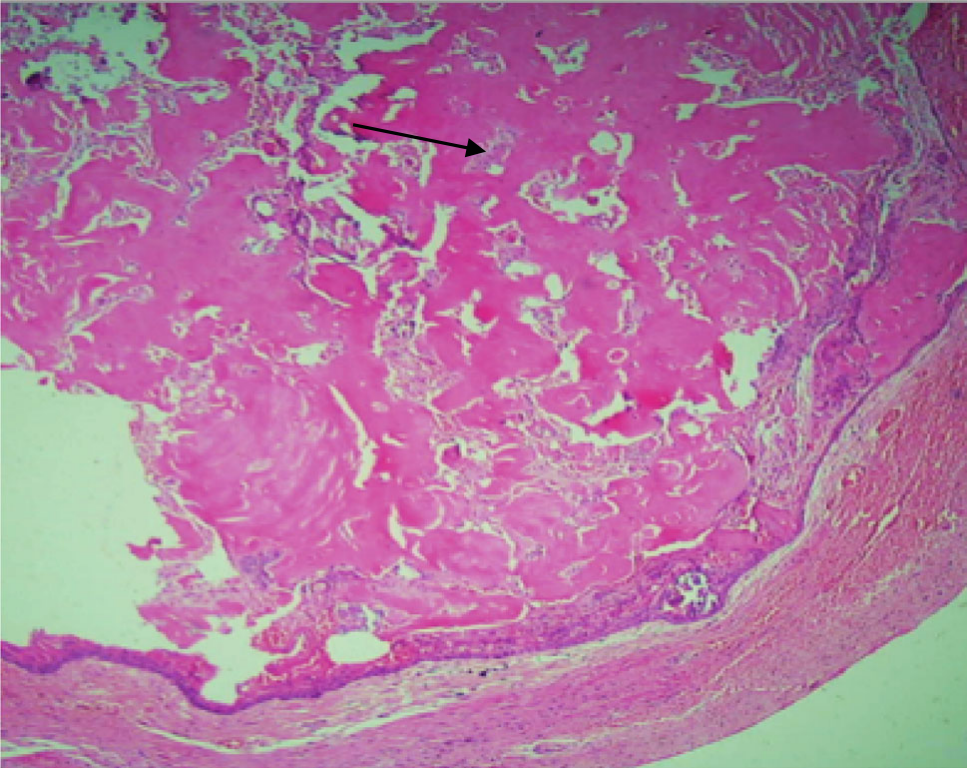
Histological section of Case 2 showing extensive dentinoid formation (arrow) (10× H&E).

**Figure 7:**
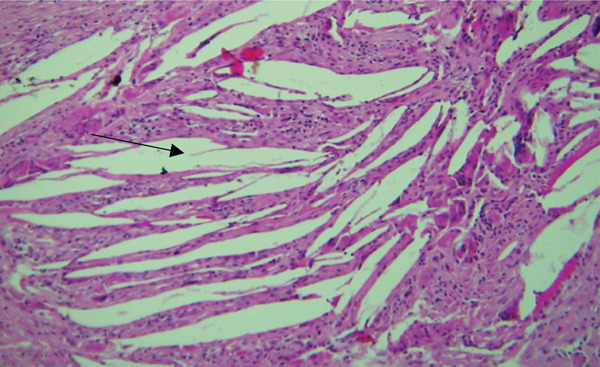
Histological section of Case 2 showing cholesterol cleft (arrow) (10× H&E).

Microscopically, the H&E–stained section showed a cystic lesion with a fibrous capsule. The basal layer of the epithelium was cuboidal to tall columnar cells with hyperchromatic nuclei, reverse polarization, and subnuclear vacuolation suggestive of ameloblastoma‐like epithelial cells (Figures 3A and 4A). The suprabasal layer showed numerous epithelial cells devoid of nuclei and eosinophilic material, and retained their basic cellular outline, suggestive of ghost cells (Figures 3B, 3C, 4B, and 5B). The focal area showed a basophilic calcified deposit (Figure 5A). The lumen was filled with extensive eosinophilic material suggestive of dentinoid (Figure 6). The area of cholesterol cleft was also noted (Figure 7).

### 3.3. Case 3

**Figure 8:**
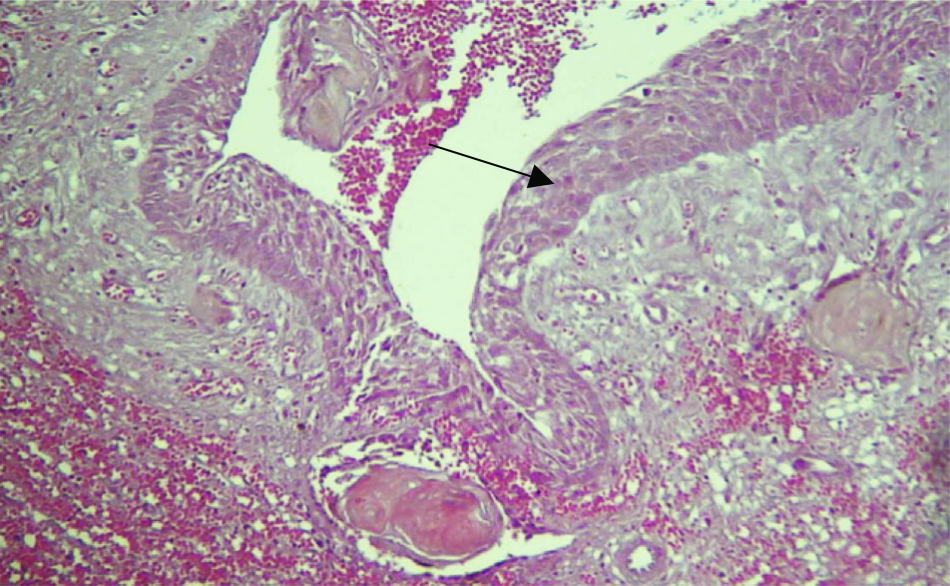
Histological section of Case 3. Arrow showing cystic lining by nonkeratinized odontogenic epithelium (10× H&E).

**Figure 9:**
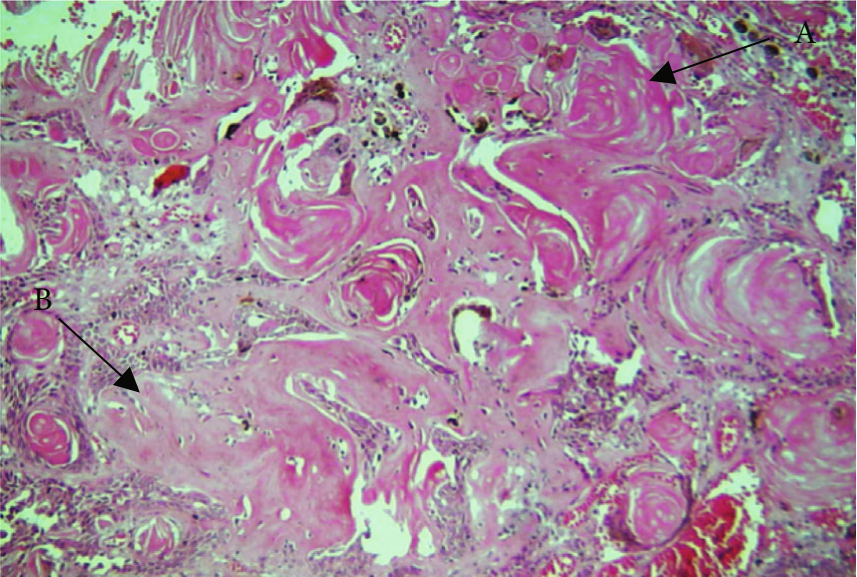
Histological section of Case 3. Arrows showing (A) ghost cells (10× H&E) and (B) dentinoid (10× H&E).

**Figure 10:**
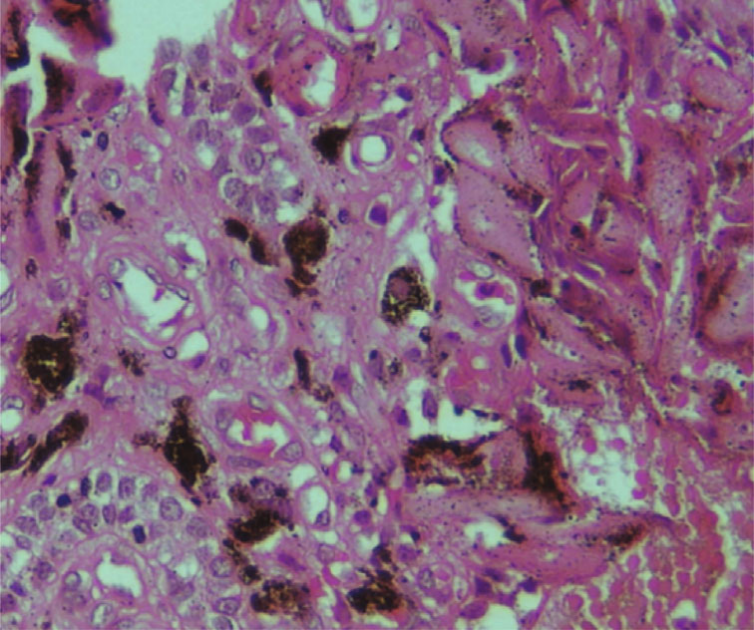
Melanin pigment within the ghost cells and odontogenic cells (40×, H&E).

Microscopically, the H&E–stained section showed a cystic lesion with a fibrous capsule and cystic cavity lined by odontogenic nonkeratinized epithelium of variable thickness (Figure 8), along with numerous eosinophilic altered cells characterized by loss of nuclei with preservation of basic cell outline suggestive of ghost cells (Figure 9A). An area of dentinoid (Figure 9B) was also evident. Melanin pigmentation is seen within the ghost cells (Figure 10).

Based on the above mentioned histopathological features of Case 1–Case 3, COCs were diagnosed.

## 4. Discussion

The COC is an uncommon developmental odontogenic cyst broadly classified as part of the family of ghost cell lesions [3]. It originated from the cell rests of the dental lamina or epithelial remnants present in the maxillary and mandibular bones or the gingival tissues. Accordingly, it can be either intraosseous (predominantly) or extraosseous [1]. The anterior jaw, including the maxilla or mandible, is the most common site for COC occurrence; however, one of our cases was evident in the posterior mandible. The essential diagnostic criteria by the fifth edition state that the lesion is mainly observed in the second to third decades of life, which again deviates from our observation, noted in the fourth, fifth, and seventh decades of life. This implies that the lesion can occur across a wide age gap. This aligns with the findings of Santos et al. [6] and Ghorbani et al. [7], and these results encourage clinicians to include COC in the common list of differential diagnoses for jaw cysts. Therefore, clinicians should not exclude COC based on the age of presentation.

COC presents as a painless swelling and is often an incidental finding in radiographic evaluation. COC radiographic presentation is usually unilocular, rarely multilocular, with well‐defined radiolucency. A tooth‐like radiopacity might also be evident within the radiolucency [1, 7]. All the presented cases showed unilocular radiolucency. The features that give a vague diagnosis, rather than being concise and hint toward the clinical diagnosis, are not confirmatory. Therefore, histopathology is not just mandatory but is diagnostic for COC.

According to WHO 2022 (fifth edition), histopathological features of COC include a unilocular cyst with a stratified epithelial lining of varying thickness, often with loosely arranged cells resembling stellate reticulum and with palisaded, hyperchromatic, columnar ameloblast‐like basal cells. All the lesions contain ghost cells, which often exhibit dystrophic calcification, and about 50% contain dentinoid in the cyst wall adjacent to the epithelium [1, 5, 8]. Cases 1 and 3 in the presented case reports show a nonkeratinized stratified epithelial lining of varying thickness. In contrast, Case 2 shows cystic lining as columnar, palisaded, hyperchromatic, ameloblast‐like basal cells with suprabasal stellate reticulum‐like cells. All the case reports show ghost cells in the cystic lining, along with calcification and dentinoid components present adjacent to the epithelium and luminal area, leading to a diagnosis of COC. These were common histopathological features as consistent findings with WHO criteria. However, the clinical diagnoses of all cases differed from the histopathological diagnosis. The clinical and radiological features that mimic other common jaw entities provide a vague diagnosis. Therefore, histopathology is essential for accurate diagnosis.

Presented Case 2 showed cystic lining of ameloblastomatous epithelium along with ghost cells and dentinoid materials in the lumen and was consistent with the histopathological description presented by Verma et al. and Shah et al. [9, 10] for ameloblastomatous COC. The author emphasized that these lesions should be differentiated from ameloblastoma arising from the lining of COC, as its behavior is governed by the ameloblastic component, which is considered aggressive. However, with the publication of the fifth edition of the WHO classification, it is clear that COC exhibits the presence of ameloblastomatous cystic lining. The presence of the ameloblast‐like lining might evoke unicystic ameloblastoma as a differential; nevertheless, the presence of ghost cells and the dentinoid component precludes it [1, 5].

All our presented case reports show ghost cells in the cystic lining, which often calcify and are essential histopathologic features of COC. Ghost cells are enlarged, ballooned, ovoid, elongated, or elliptoid epithelial cells with a well‐defined cellular outline, eosinophilic cytoplasm, and a faint outline of the nucleus or no nucleus. The origin of ghost cells has been linked to coagulative necrosis, enamel protein deposition, or abnormal keratinization. However, histochemical studies have supported abnormal keratinization as most justified, as they stained positively with keratin staining dye, like Mallory and Van Gieson stains [1]. Ghost cells also tend to fuse and form large sheets of amorphous and acellular materials, as evident in our two cases. Ghost cells might extrude through the epithelium into the adjacent connective tissue, initiating a foreign body giant cell reaction, or may show dystrophic calcification [1, 5]; both of which are evident in our cases.

All our cases exhibit extensive dentinoid within the cystic lumen. The focal presence of dentinoid components is common and might occur in 50% of cases, but this is not considered an essential feature of COC. Limited literature exists on extensive dentinoid formation. The studies conducted by Santos et al. [6], Menditti et al. [11], and Fregnani et al. [12] show extensive dentinoid formation in both lumen and connective tissue. The literature describes dentinoid as resulting from the inductive effect of odontogenic epithelium on adjacent mesenchymal tissue. However, the precise role of extensive dentinoid formation remains unclear. It may be related to the aging of the lesion and its potential transformation into the solid variant, such as dentinogenic ghost cell tumor (DGCT). It may be related to the aging of the lesion and its potential transformation into the solid variant, such as DGCT. Diagnostic challenges are due to the presence of extensive dentinoid and ghost cells, which share similar features as a DGCT; however, the presence of a cystic lumen, unilocular lesion, absence of extensive ameloblastomatous islands, and dentinoid within connective tissue excludes DGCT in our cases. DGCT often appears as multicystic, and during gross examination, opening the cyst cavity reveals a solid lesion, accompanied by extensive ameloblastomatous islands or dentinoid within the connective tissue [1, 5].

Multinucleated giant cells were seen in one of our cases (Case 1), the ghost cell extruding from the epithelium into the adjacent connective tissue and which may elicit the foreign body reaction. The finding reported in the literature by Hsu et al. explained that the multinucleated giant cells were observed due to foreign body reaction [1, 5, 13].

Evidence of an odontogenic epithelial rest cell island was present in developmental cysts of the jaw and was reported in nine out of 13 cases in the case series by Takeda et al. and was consistent with our observation as well [13].

The presence of cholesterol clefts was noted in Case 2, which may be an unusual presentation for an uninfected odontogenic cyst like COC and could indicate degenerating cells. Cholesterol clefts in radicular cysts have been linked to disintegrating red blood cells, bacterial products that readily crystallize within the tissues. In our cases, cholesterol could be derived from degenerating red blood cells, lymphocytes, plasma cells, and macrophages. However, limited literature is available regarding cholesterol clefts in uninfected COC [14].

Melanin pigments were observed in ghost cells in one of our case reports (Case 3); this feature is uncommon in COCs. The study conducted by Han et al. revealed that variable amounts of melanin were found in the epithelial lining, including within ghost cells [15]. The mechanisms by which melanocytes and melanin pigment appear in odontogenic cysts remain unclear. Some researchers suggest that the neural crest influences the development of odontogenic tissues and the occurrence of melanocytes in these tissues. Others propose that melanin production might be associated with racial pigmentation. A biochemical study showed that the level of free radical concentration is increased in cysts of the jaw; melanin acts as a scavenger of free radicals. The presence of inflammatory cells can trigger melanogenesis, which reduces the inflammatory response [16]. The presence of melanin pigments and scattered inflammatory cells could be the cause in our case. Further studies are necessary to explain the impact on COC and the exact etiopathogenesis of melanin.

In immunohistochemistry, Cytokeratins 14 and 19 have been detected in the epithelial component, with CK6 expression in both the epithelium and ghost cells of COC [17]. The study by Takata et al. showed that the cytoplasm of ghost cells in COCs expressed enamel‐related proteins (amelogenin, enamelin, enamelysin, and sheathlin), while ghost cells in the calcifying epitheliomas (Malherbe) of the skin were negative [18]. Ahn et al. suggest a role for *β*‐catenin and the WNT signaling pathway in the development of COCs [1]. Similarly, a study by Sekine et al. found somatic *β*‐catenin mutations in 9 of 10 COCs analyzed [19].

Intraosseous/central COCs are treated by enucleation. Conservative surgical excision and marsupialization, followed by enucleation, are the other techniques attempted based on the size and location of the lesion [8]. Two of our cases (Case 2 and Case 3) underwent surgical enucleation with no recurrence to date. However, Case 1, which was planned for marsupialization followed by enucleation based on the size extending from mandibular jaw 34–44, was lost to follow‐up. Early recognition and appropriate treatment often result in an excellent prognosis, despite follow‐up being mandatory to avoid potential recurrences. The overall recurrence rate is less than 8% for both intraosseous and extraosseous COCs, and the long‐term prognosis is good [5].

## 5. Conclusion

The clinical diagnosis of all the reported cases was different from the histopathological diagnosis. In the absence of a characteristic clinical and radiographic presentation, histopathology stands to be the gold standard for diagnosing COC. Reporting these cases contributes to the scientific literature by emphasizing the prevalence of such uncommon entities with diverse histopathological findings. Despite the change in the histopathological diagnostic criteria as per the classification system, every histopathological aspect signifies the nature and behavior of the lesion and is important to interpret for the management.

## Consent

Written consent was obtained from the patients.

## Conflicts of Interest

The authors declare no conflicts of interest.

## Author Contributions

Saraswoti Khadka: manuscript preparation, data collection, and editing. Toniya Raut: manuscript editing. Shashi Keshwar: manuscript editing. Neetu Jain: manuscript review. Ashish Shrestha: manuscript review. Mehul Rajesh Jaisani: manuscript review. Khushbu Adhikari: manuscript review.

## Funding

This study did not receive any funding in any form.

## Data Availability

The data that support the findings of this study are available from the corresponding author upon reasonable request.
